# Nerve detection with optical spectroscopy for regional anesthesia procedures

**DOI:** 10.1186/s12967-015-0739-y

**Published:** 2015-12-15

**Authors:** Benno H. W. Hendriks, Andrea J. R. Balthasar, Gerald W. Lucassen, Marjolein van der Voort, Manfred Mueller, Vishnu V. Pully, Torre M. Bydlon, Christian Reich, Arnold T. M. H. van Keersop, Jeroen Kortsmit, Gerrit C. Langhout, Geert-Jan van Geffen

**Affiliations:** In-Body Systems Department, Philips Research, High Tech Campus 34, 5656AE Eindhoven, The Netherlands; Department of Anesthesiology and Pain Medicine, Maastricht University Medical Center, Maastricht, The Netherlands; Philips Healthcare, Best, The Netherlands; Department of Surgery, Netherlands Cancer Institute, Amsterdam, The Netherlands; Department of Anesthesiology, University Medical Center St. Radboud, Nijmegen, The Netherlands

**Keywords:** Regional anesthesia, Nerve detection, Diffuse optical spectroscopy, Classification methods

## Abstract

**Background:**

Regional anesthesia has several advantages over general anesthesia but requires accurate needle placement to be effective. To achieve accurate placement, a needle equipped with optical fibers that allows tissue discrimination at the needle tip based on optical spectroscopy is proposed. This study investigates the sensitivity and specificity with which this optical needle can discriminate nerves from the surrounding tissues making use of different classification methods.

**Methods:**

Diffuse reflectance spectra were acquired from 1563 different locations from 19 human cadavers in the wavelength range of 400–1710 nm; measured tissue types included fascicular tissue of the nerve, muscle, sliding fat and subcutaneous fat. Physiological parameters of the tissues were derived from the measured spectra and part of the data was directly compared to histology. Various classification methods were then applied to the derived parameter dataset to determine the accuracy with which fascicular tissue of the nerve can be discriminated from the surrounding tissues.

**Results:**

From the parameters determined from the measured spectra of the various tissues surrounding the nerve, fat content, blood content, beta-carotene content and scattering were most distinctive when comparing fascicular and non-fascicular tissue. Support Vector Machine classification with a combination of feature selections performed best in discriminating fascicular nerve tissue from the surrounding tissues with a sensitivity and specificity around 90 %.

**Conclusions:**

This study showed that spectral tissue sensing, based on diffuse reflectance spectroscopy at the needle tip, is a promising technique to discriminate fascicular tissue of the nerve from the surrounding tissues. The technique may therefore improve accurate needle placement near the nerve which is necessary for effective nerve blocks in regional anesthesia.

## Background

**Fig. 1:**
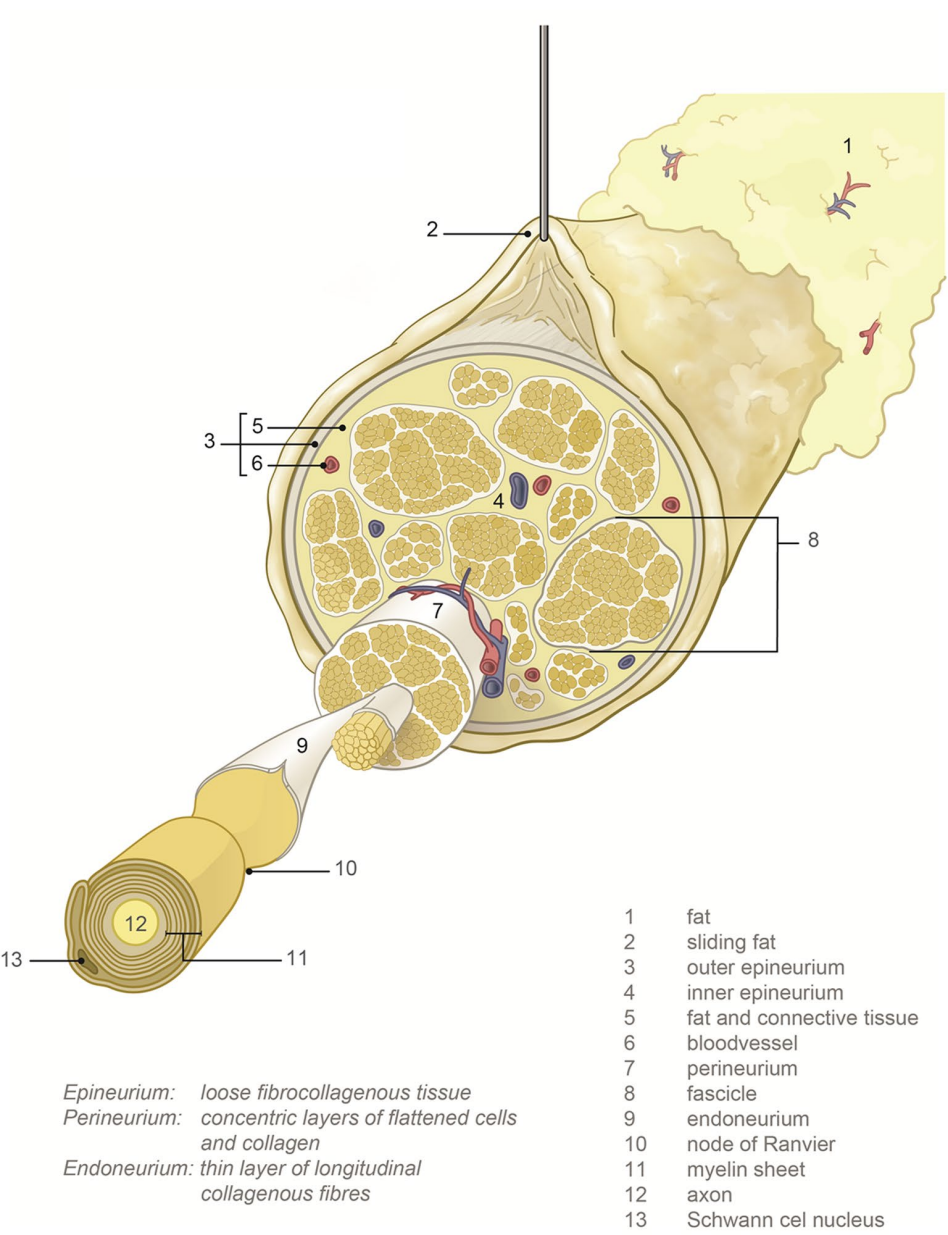
Schematic drawing of the nerve and the surrounding tissues

Regional anesthesia has shown advantages over general anesthesia; lower odds of inpatient mortality and pulmonary complications during hip repair [1], faster return of bowel function [2] as well as a lower risk for delirium and nausea have been reported. Current disadvantages of regional anesthesia are the unpredictable onset time of the block as well as the risk of a failed block and, although rare, the risk for complications caused by injection of local anesthetics into critical structures such as blood vessels or nerve fascicles (see Fig. 1) [3, 4]. Accurate needle placement with respect to the various structures is therefore important [5].

Techniques like paresthesia, electrical nerve stimulation and ultrasound guidance have been introduced to improve needle placement. The elicitation of paresthesia has shown a sensitivity of only 40 % for nerve detection while for nerve stimulation up to 75 % sensitivity has been reported [6, 7]. However, the stimulation threshold does not give an exact indication of the distance between the needle and nerve. Ultrasound (US) guidance for the performance of peripheral nerve blocks improves the quality of regional anesthesia because nerve structures can be seen as multiple rounded hypoechoic areas in a hyperechoic background or vice versa [8] and the spread of local anesthetic is observed upon injection. However, the quality of the ultrasound guidance is user- and patient-dependent, requires sufficient training, and additionally ultrasound imaging has technical limitations. For example, as the US frequency decreases, degradation in the image resolution occurs [9].

Diffuse optical spectroscopy is a technique which sends white light into tissue and measures the reflected light. The reflected light has a specific spectral distribution due to the absorption and scattering of photons interacting with the tissue. Since these changes are determined by the tissue composition, the technique provides tissue discrimination. By incorporating optical fibers into needles, a method has been developed that can provide real-time tissue feedback at the tip of the needle. This spectral tissue sensing (STS) method is complementary to existing techniques that do not have this functionality. Therefore, STS could improve needle placement in regional anesthesia for accurate deposition of anesthetics. Recently, several studies have been performed aiming at nerve discrimination based on diffuse optical spectroscopy [10–12]. In the study of Brynolf et al. [10] the optical detection of the brachial plexus was studied in in vivo swine. While promising results were presented, the study was limited to detecting a “nerve target region” and performed on only two swine. Balthasar et al. [11] investigated optical detection of peripheral nerves in 20 patients. Subcutaneous fat, muscle and a nerve target region were the tissue types included and detected in the study. The results indicated that the transitions from muscle to nerve target region could be identified reliably. However, the question of nerve proximity remained unanswered. Differentiation between nerve and adipose tissue with optical spectroscopy was investigated in thyroid and parathyroid surgery by Schols et al. [12]. Discrimination between nerve and adipose tissue was possible especially when measuring the spectra in the near infrared region. However, due to the limited dataset of only including adipose tissue and nerve, no definitive conclusions could be drawn from this data regarding needle placement for regional anesthesia where the needle also traverses subcutaneous fat and muscle tissue.

In this study it was investigated whether accurate needle placement near nerve structures of the cervical nerves (C-roots) and the median nerve of human cadavers was possible when guided by STS to facilitate needle placement for regional anesthesia. Furthermore, to investigate whether a detection algorithm based on fascicular tissue of the cervical nerve can be generalized to other nerves, spectra measured at different locations near the median nerve was validated on a model trained on the spectra acquired from the cervical nerve area.

## Methods

### Human specimens

Human tissue measurements were performed at the Anatomy department of the Radboud University Medical Center Nijmegen. In this study STS measurements were performed in 19 human cadavers, 11 male and 8 female with an age range of 65–92 years (mean age 80.4 years). A handwritten and signed codicil from the donor, as required by the Dutch law for scientific research and education is kept at the Department of Anatomy of the Radboud University Nijmegen, Nijmegen, The Netherlands.

The human cadavers were one-time frozen and selected to have no neurological abnormalities or diseased tissue near the intended measurement sites. In each cadaver the cervical area (from interscalene region to proximal until the neuroforamen) and forearm area (medial side of the mid forearm) was dissected to expose the nerves and surrounding tissues. In all human cadavers cervical nerves and surrounding tissues were measured and in ten of them the median nerves with surrounding tissues were also measured.

### Cervical nerve, median nerve and surrounding tissue anatomy

**Fig. 2:**
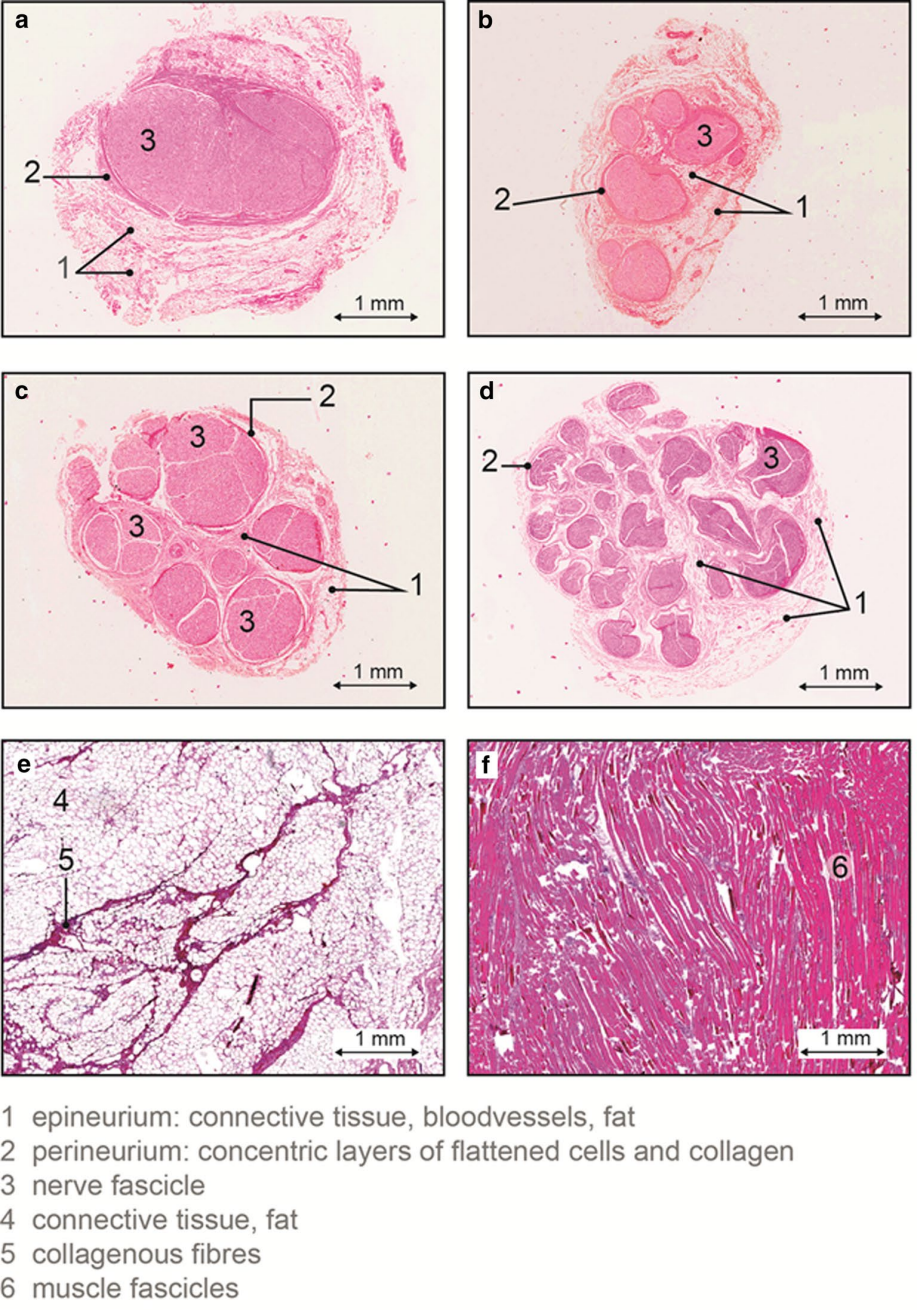
Histology slides of the nerves and surrounding tissues studied: cross section of the cervical nerve (C6-root) with some surrounding areolar tissue proximal (**a**) and distal (**b**); cross section of the median nerve proximal (**c**) and distal (**d**), subcutaneous fat (**e**) and muscle (**f**)

In general after skin perforation, when advancing the needle towards the nerve a layer of subcutaneous fat, muscle tissue and the fat surrounding the nerve (sliding fat) will be traversed (see Fig. 1). Sliding fat in our case is defined as fat surrounding the entire nerve bundle; fat outside the outer epineurium. In Fig. 2 the histology of subcutaneous fat, muscle, the cervical nerve and the median nerve is shown. The cervical nerve at the proximal side is mostly mono-fascicular branching into multiple fascicular bundles at the distal side. The fascicular bundles are surrounded by loose collagenous tissue and adipose tissue of the epineurium [13]. The median nerve is already multi fascicular at the proximal side, with the number of fascicles increasing towards the distal side. Subcutaneous fat shows a high amount of adipose cells with some connective tissue while muscle tissue is mostly composed of muscle fibers.

### Instrumentation

**Fig. 3:**
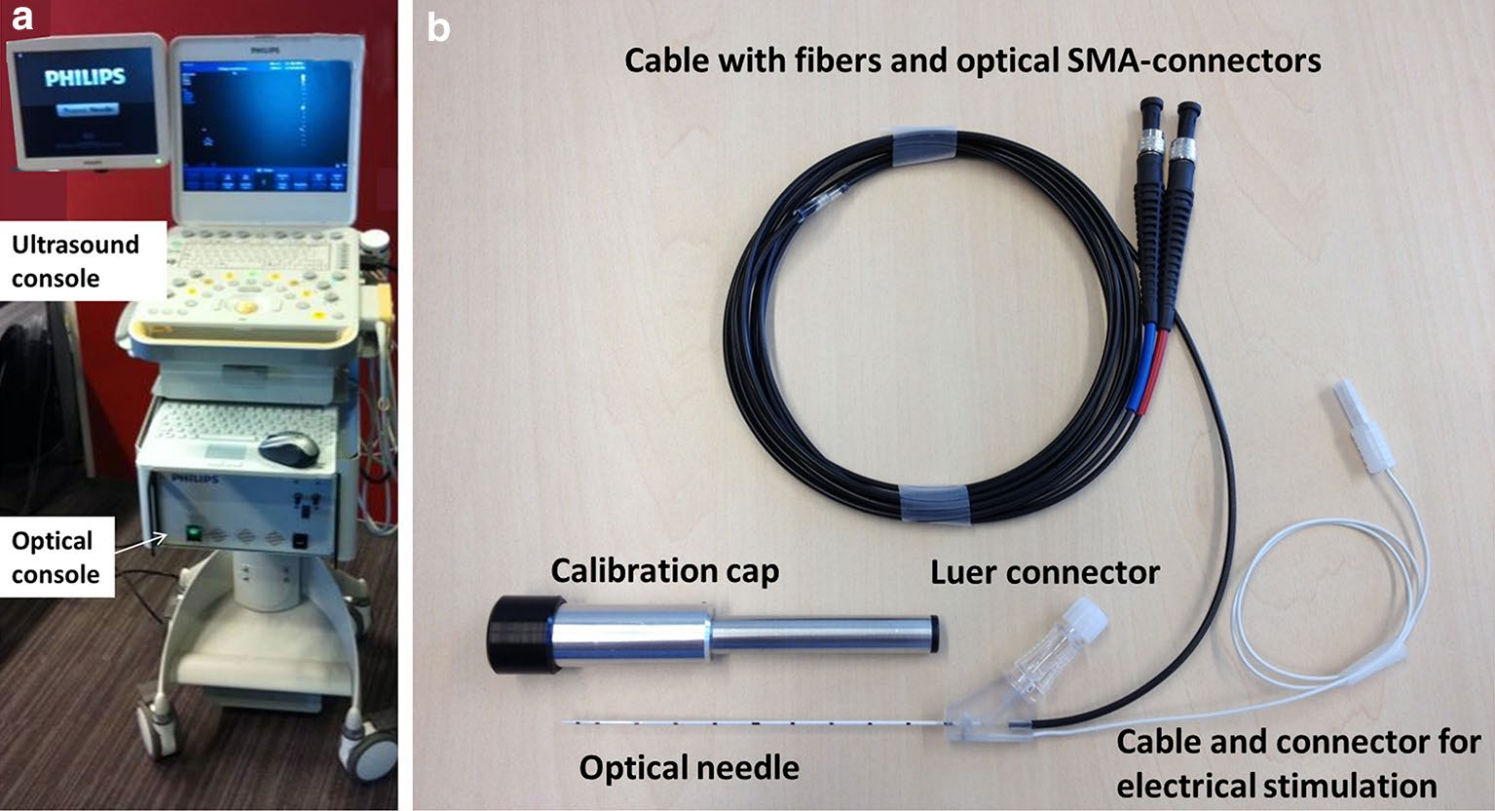
Picture of the optical console (*left*) and the optical needle (*right*) used in the measurements

Ex vivo diffuse reflectance spectra were acquired using a portable spectroscopic system as illustrated in Fig. 3. A tungsten halogen broadband light source with an integrated shutter (Avantes HAL-S) was used as an emitting source. Delivery of light to the tissue and its collection were achieved with a 1.0 mm diameter fiber-optic probe with the distal end polished at an angle of 30 degrees. The probe consisted of two 200-μm core diameter optical fibers with a tip to tip distance of 0.85 mm. The illumination fiber was connected to the light source and the collection fiber was connected to a spectrometer with a silicon detector (Horiba, S318-2 VIS) and a spectrometer with an InGaAs detector (Horiba-S330-2 NIR), via a 50–50 % fiber splitter to divide the collected light over the two detectors.

The diffuse optical spectra were calibrated by subtracting a background measurement acquired after each tissue measurement and divided by the response of a white reflectance standard (Spectralon) with known reflectivity.

Acquisition time per spectrum was 1 s including background measurement. At each measurement location typically 10 spectra were acquired that were averaged to form one data point in the database.

### Data acquisition

In this study STS measurements were performed on 19 human cadavers. A database was compiled of spectra measured at 1274 different locations on and near nerve roots as well as at 289 different locations on and near the median nerve. Spectra (400–1710 nm) were acquired from subcutaneous fat, muscle, sliding fat, and fascicular tissue of the nerve. Two experienced anesthesiologists, with the assistance of an anatomist, positioned the optical needle on the exposed tissues. In all cases we ensured that the optical fibers of the needle made good contact with the tissue to avoid artifacts due to air voids. Because the needle was held by hand some slight movements could have occurred but analysis of several measurements taken at one location did not show significant variations in the spectra. To avoid that a mixture of the tissue types was measured we made sure that the tissue layer was thicker than 2 mm. Especially for sliding fat, the optical needle was placed such that no nerve structure was in the direction of the light beam.

### Feature extraction methods

All data processing was performed using software developed in-house based on MATLAB (Mathworks Inc.) and its Statistics toolbox. Where third party toolboxes were used, they are mentioned below.

With a resolution of 1 nm each measured spectrum consists of 1311 data points. To avoid over-fitting, the number of data points must be translated into features, with the number of features considerably lower than the number of spectra measured. Several methods to reduce the number of data points into relevant features were considered. In particular we considered methods with limited dependence on absolute intensities since the absolute intensity of the spectra may fluctuate significantly due to small variations in calibration conditions, the state of the probe, or even the bending radius of the fibers.

The first feature extraction method was a fitting method which decomposes a spectrum into 12 fit parameters i.e. features. The second method was to perform a standard principal component analysis (PCA) and use the most important principal components as features [14].

#### Physiological fit parameters

Spectral data modelling can relate the measured spectra to underlying physiological parameters like blood and fat concentrations. The spectra were fit with a modified version of the model developed by Farrell et al. [15]. This model estimates the absorption coefficient $\upmu_{\text{a}} \left(\uplambda \right)$ and the reduced scattering coefficient μ_s′_(λ) expressed in cm^−1^. To determine the chromophore concentrations present in the tissue, the fiber distance between the emitting and collecting fibers as well as the wavelength-dependent absorption coefficients of these chromophores need to be known [16–18]. The chromophores that were taken into account in this study were the blood related absorbers deoxygenated-hemoglobin (Hb) and oxygenated-hemoglobin (HbO2), β-carotene, fat, water and collagen. The blood oxygen saturation (StO2) is defined as the fraction of hemoglobin that is oxygenated i.e. HbO2/(Hb + HbO2). The fat-to-water and fat fraction F/(W + F) is defined as fat content divided by the water and fat content of the tissue. For the scattering the empirical model described by

1 $$\upmu_{{\text{s}}{^{\prime}}} \left(\uplambda \right)\text{ = S800}\left[ {\text{Fmie}\left( {\frac{\uplambda}{{\uplambda_{\text{0}} }}} \right)^{ -{\text{ b}}} + \left( 1{ - } {\text{Fmie}} \right)\left( {\frac{\uplambda}{{\uplambda_{\text{0}} }}} \right)^{ - {\text{4}}} } \right]$$

is used where $\uplambda_{0}$ = 800 nm corresponds to a wavelength normalization value, S800 is the reduced scattering amplitude at λ_0_, the Mie scattering slope is b, and Fmie denotes the Mie-to-total reduced scattering fraction assuming that Mie and Rayleigh scattering are the two dominant types of scattering in tissue.

#### Wavelength dependent features

Each fit parameter and principal component relates to the full wavelength range of the spectrum, so we also developed a method whereby each feature relates to a certain wavelength range. In this third method the spectrum was divided into equally-sized wavelength segments. For each segment a feature was calculated according to how the spectrum slopes in the segment as compared to the averaged spectra. To avoid the influence of intensity variations this was performed on normalized spectra. To determine the difference from the average spectra, the intensities were scaled for each wavelength, so that the average intensity was 0 and the standard deviation was 1. The scaled intensities were then added to obtain a scalar feature. Segment values are strongly positive when a spectrum slopes significantly more upward than the average, strongly negative when it slopes more downwards, and around zero when it behaves averagely or when the individual spectrum crosses the average spectrum.

This segment feature extraction method was considered as representative for the many other methods that can be used to describe the (relative) behavior of a curve.

### Classification methods

Different classification methods were investigated to discriminate between fascicular tissue of the nerve and non-fascicular tissue (muscle, subcutaneous and sliding fat). These included: partial least squares discriminant analysis (PLS-DA), support vector machine (SVM) and classification and regression trees (CART) analysis.

#### Partial least squares discriminant analysis (PLS-DA)

Partial least-squares (PLS) [19] analysis is a regression method to find a linear relationship between a response variable Y (tissue type class) and the independent variable X (spectra). PLS-DA is widely used for analysis of spectra. The method is based on finding a number of principal components that represent as much of the variance in X as is possible. PLS-DA selects the principal components which are most relevant to the response variable Y. Therefore, PLS-DA acts directly on the spectra without the need for prior feature extraction. The PLS model is generated using a training data set. A discriminant analysis (DA) method is subsequently performed to obtain thresholds for discriminating the different responses (i.e. tissue classes). Prediction of class (tissue type) on the remaining data (the validation data set) is obtained by comparing the predicted PLS scores with the DA thresholds. The measured tissue type is assigned to one of the two predefined tissue classes depending on the PLS scores. The PLS-DA algorithm scripts used PLS Toolbox 6.2 (Eigenvector Research, Inc, Wenatchee, WA).

#### Support vector machine (SVM)

Support vector machines (SVM) [20] divide the feature space with a hyperplane that separates the two classes. In case full separation is not possible, a penalty parameter determines the behavior of the boundary. We used LIBSVM (http://www.csie.ntu.edu.tw/~cjlin/libsvm/) with a radial Kernel and standard parameters. Before using SVM, all features were scaled to a mean of 0 and a standard deviation of 1.

#### Classification and regression trees (CART) analysis

The classification and regression tree (CART) is based on a binary recursive partitioning algorithm. It starts from a central node that discriminates the two classes based on the best discriminating feature. From this node, daughter partial trees are generated and other features or the same are used for further splits. The purity of each node is assessed with the Gini’s maximization index algorithm which corresponds to unity minus the sum of square of the proportions of target classes at a specific node [21].

#### Statistical analysis

All classification methods used a training data set to create a classification model. A second, validation data set was then used to calculate accuracy, sensitivity and specificity for the model based on the confusion matrix of the validation data. Sensitivity and specificity are complementary (one can improve sensitivity at the cost of specificity and vice versa) so Matthews correlation coefficient (MCC) was used as a scalar measure for the quality of classification. MCC is given by [22]:

2 $$MCC = \frac{TP \times TN - FP \times FN}{{\sqrt {(TP + FP)(TP + FN)(TN + FP)(TN + FN)} }},$$

where TP are the true positives, TN the true negatives, FP the false positives and FN the false negatives. Here, fascicular tissue of the nerve was considered as a “positive” and surrounding non-fascicular tissues as a “negative”. The value of MCC is between −1 and 1, where 1 represents a perfect prediction, 0 no better than random and −1 total disagreement between prediction and actual state.

For the cervical nerve dataset a leave-one-specimen-out cross-validation approach was used, meaning the classification models were trained on the spectra from all but one cadaver and the validation was done on the spectra from the cadaver left out. This was repeated until all cadavers had been left out once, with the confusion matrices being added up.

The datasets acquired for the median nerve and surrounding tissue were classified by training the algorithms on the cervical nerve and surrounding tissues database only and then applying the model to the datasets collected in the area of the median nerve. This was to show how the classification algorithms work on truly independent data.

## Results

### Tissue spectra

In total spectra were collected from 1274 different locations in the neck area of 19 human cadavers resulting in a dataset consisting of: 702 fascicular measurements from the cervical nerves, 223 muscle, 164 sliding fat and 185 subcutaneous fat. In addition, spectra were collected from 289 different locations near the median nerve: 107 median nerve, 76 muscle, 46 sliding fat and 60 subcutaneous fat tissues.

**Fig. 4:**
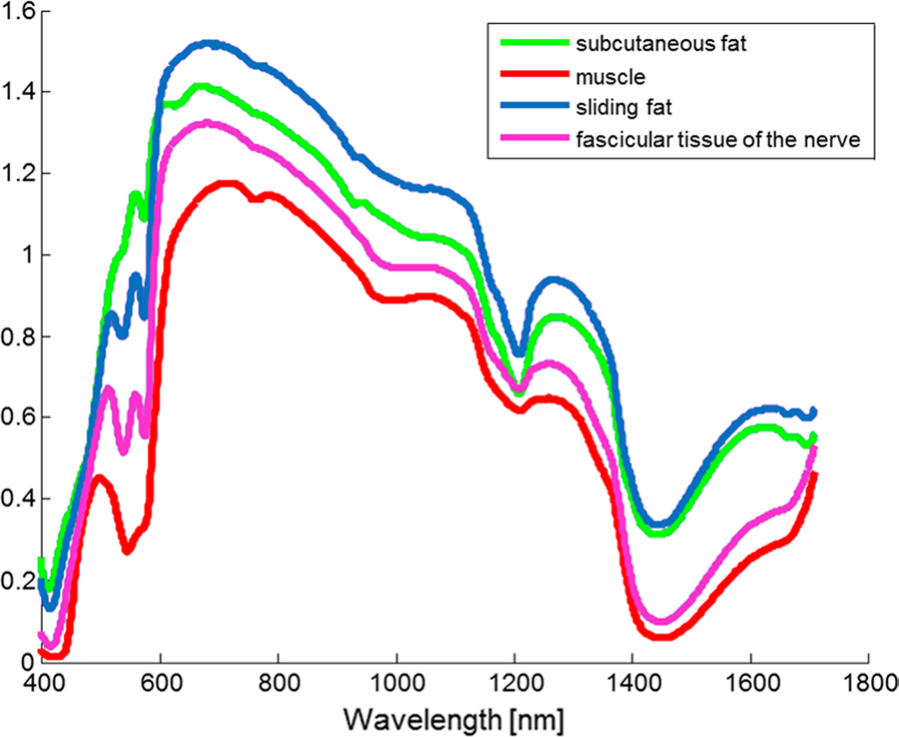
Averaged spectra measured for the four tissue classes

**Fig. 5:**
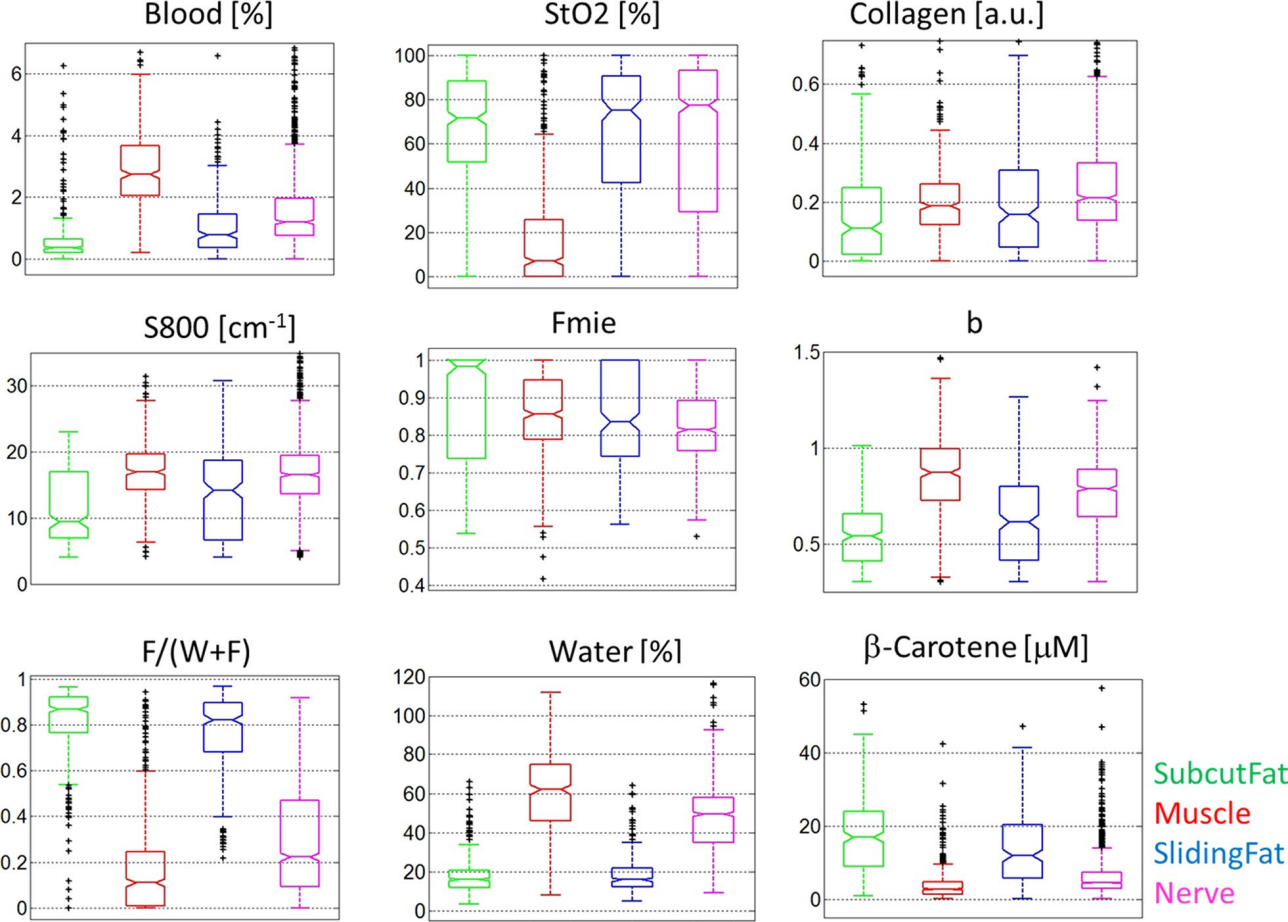
*Boxplots* of the various parameters derived from the fit model. Note the *plots* have been adjusted to maximize visualization of the *boxes* and *whiskers*; therefore not all outliers are shown

In Fig. 4 the average spectra are shown measured on subcutaneous fat, muscle, sliding fat and fascicular tissue of the cervical nerve (nerve tissue). To determine the physiological differences between the tissues in Fig. 5 boxplots of the most relevant parameters determined by the fit model are shown. A blood content of 100 % is equivalent to 150 g hemoglobin/liter. The blood content in muscle tissue is found to be higher than nerve, and is lowest in subcutaneous and sliding fat which is in line with expectations based on the diffuse reflectance spectrum shown in Fig. 4, since the amount of blood is linked to the absorption dip near 550–600 nm. Blood oxygen saturation was low for muscle tissue and comparable for the other tissues. Adipose rich tissues (subcutaneous and sliding fat), show high fat content, while for nerve and muscle lower values were found. This is also observed in the typical spectra where higher fat values result in a more pronounced absorption dip around 1210 nm. Beta-carotene, absorbing near 500 nm causing a yellow appearance of tissue, shows a similar behavior to the fat content. Results for the collagen content estimated from the fit model show higher values in muscle and nerve. For the scattering related parameters, the contribution of the Mie scattering (Fmie) and the Mie slope parameter (b) show largest differences between the four tissues.

### Tissue classification

In Table 1, the classification results for fascicular discrimination from the surrounding non-fascicular tissues (subcutaneous fat, muscle and sliding fat) according to the methods considered in this paper are summarized. For the SVM classification, the different feature extraction methods (Fit, PCA and Segments) as well as a combined feature that combines all three were considered. The classification results show that accurate discrimination of nerve tissue is possible, independent of the classification method used. The highest achieved sensitivity and specificity were both around 90 %.

**Table 1:** Classification results according to SVM, PLS-DA and CART for discrimination of fascicular tissue of the nerve from the surrounding tissues

Classification method	Feature selection	MCC	ACC	SENS (%)	SPEC (%)	PPV	NPV	TP	FN	FP	TN
SVM	Fit	0.711	0.854	82.6	88.8	0.901	0.806	580	122	64	508
SVM	PCA	0.793	0.897	89.9	89.5	0.913	0.878	631	71	60	512
SVM	Segments	0.779	0.890	88.6	89.5	0.912	0.865	622	80	60	512
SVM	Combined	0.826	0.914	91.3	91.4	0.929	0.896	641	61	49	523
PLSDA	10PC’s	0.814	0.907	92.5	89.5	0.864	0.943	494	40	78	662
CART	Fit	0.615	0.808	81.2	80.4	0.836	0.777	570	132	112	460

For SVM different feature selection methods are used: fit parameters, PCA, segments and a combination of the last three. For PLSDA, 10 principal components have been used (10PC’s). For the CART analysis, the fit parameters have been used as features

Matthews correlation coefficient (MCC see Eq. 2 text), accuracy (ACC = [TP + TN]/[TP + FN + FP + TN]), sensitivity (SENS = TP/[TP + FN]), specificity (SPEC = TN/[FP + TN]), positive predictive value (PPV = TP/[TP + FP]), negative predictive value (NPV = TN/[TN + FN]), true positive (TP), false negative (FN), false positive (FP), true negative (TN)

The number of principal components and the length of the segments were optimized in a coarse way. For SVM, 30–40 principal components and short segments with a length between 10 and 20 nm resulted in the best performance. The table contains the values for 30 principal components and 20 nm segments. As mentioned before, a leave-one-specimen-out cross-validation approach was applied for this analysis.

To investigate the validity of the classification when applying the models trained on the data from the neck area to classify spectra from tissues in other areas of the body, the above analysis was repeated. In Table 2, the classification results are listed for median nerve discrimination from the surrounding tissues when the classification algorithm was trained on data collected from C-root nerves and surrounding tissues in the neck area only. Overall, the results of that analysis show somewhat lower sensitivity and specificity. Nonetheless, sensitivity and specificity above 85 % were achieved with most methods.

**Table 2:** Classification results according to SVM, PLS-DA and CART for discrimination of fascicular tissue of the median nerve from the surrounding tissues validation data collected in the forearm area) when training the various methods using data collected in the neck area

Classification method	Feature selection	MCC	ACC	SENS (%)	SPEC (%)	PPV	NPV	TP	FN	FP	TN
SVM	Fit	0.683	0.848	85.0	84.6	0.765	0.906	91	16	28	154
SVM	PCA	0.693	0.855	83.2	86.8	0.788	0.898	89	18	24	158
SVM	Segments	0.717	0.862	88.8	84.6	0.772	0.928	95	12	28	154
SVM	Combined	0.751	0.882	86.9	89.0	0.823	0.920	93	14	20	162
PLSDA	10PC’s	0.580	0.789	84.1	75.8	0.672	0.890	90	17	44	138
CART	Fit	0.442	0.730	71.0	74.2	0.618	0.813	76	31	47	135

## Discussion and conclusion

In this study the accuracy with which STS is able to discriminate nerve tissue from surrounding tissues during needle interventions for regional anesthesia was investigated. Of four tissue types spectra were collected from 19 human cadavers at 1563 different locations: 1274 spectra acquired from the cervical nerve area and 289 spectra from the median nerve area. Various classification algorithms were applied to the dataset showing that fascicular tissue of the nerve can be discriminated from surrounding tissues with sensitivity and specificity around 90 %. These results were based on the largest dataset collected to date in nerve and surrounding tissues and confirm earlier reports on nerve discrimination with smaller datasets [10–12].

The histology slides show that the nerves studied consisted mainly of fascicular bundles enclosed by loose collagenous tissue and adipose tissue of the epineurium. The outer-epineurium is surrounded by a layer of sliding fat with variable thickness. The boxplots of the physiological parameters obtained by fitting the spectra show a significant difference between fat content for tissues such as subcutaneous fat and sliding fat compared to muscle and nerve tissues. The same holds for beta-carotene which is present in fatty tissues [18]. The blood content in muscle tissue is on average higher while the blood oxygen saturation herein is lower than in the surrounding tissues. Furthermore, from the parameters related to scattering (S800, Fmie and b), the Mie slope b seems to differ most between the tissues. Collagen content shows higher values in muscle and nerve which is in line with the fact that those tissues are richer in connective tissue compared to the other tissues investigated [13]. In general, there is a significant variation in the parameters determined within each tissue, and outliers are present (indicated with ‘+’). Despite these variations and outliers, the nerve tissue can be discriminated with high accuracy from the surrounding tissues for this large data set.

The spectra were measured in one-time frozen human cadavers, where the tissues were exposed so that the physician was able to validate the tissue type measured visually. Physiological parameters that are known to change from in vivo to ex vivo are the blood content and blood oxygen saturation. The settling of blood (livor mortis [23]) will affect the local blood content while the blood oxygen saturation in the exposed tissue is likely to be different from living tissue because of no blood circulation as well as the uptake of oxygen directly from the air. In what way this affects the nerve discrimination when performing the measurements in vivo is part of future investigation.

Of the various dimension reduction methods the fit method resulted in the lowest MCC. The fit method reduces the spectral information into 12 independent parameters thereby losing relevant information for discriminating nerves. Additionally the fit method, based on the analytical model derived by Farrell et al. [15], assumes the tissue to be homogenous. The histological results, however, show that within the optical probing volume of ~1 mm^3^ this assumption is not always fulfilled, especially near the nerve that has a more layered structure. Despite these disadvantages, the fit method provides the opportunity to link observed features in the spectra to the histological and physiological properties of the studied tissues. Trends in the boxplots of Fig. 5 can be linked to the histology shown in Fig. 2.

There is significant variation present in the measured spectra per tissue type. As a result, discrimination of the various tissues requires taking multiple parameters into account, in our case up to 40 spectral features. The danger of over fitting the data is prevented by our large spectral database collected in 19 specimens. From the classification methods, SVM performed best. SVM may be able to perform well on multi-dimensional parameter spaces because it can ignore features that do not significantly contribute to the discrimination. The fit dimension reduction allows easy interpretation of the results since the input parameters for classification are used whereas other methods post-process the parameters into scores that might not be intuitively related to the input parameters. From the fit parameters, the scattering parameters, the fat and water content as well as blood oxygen saturation contributed most to the fascicular tissue of the nerve discrimination. In general, the various classification methods resulted in similar accuracies, demonstrating the robustness of STS for tissue discrimination. This robustness allows flexibility in classification algorithm design when developing tissue discrimination based on STS into a product.

The cervical nerves as well as the median nerves investigated in this study are mostly composed of fascicular tissue with a limited amount of epineurium that consists of irregular connective tissue and adipocytes. This composition is known to be different in various nerves and changes when going from proximal to distal locations of the nerve [24]. When training the classification algorithm on the cervical nerve area only and testing it on the median nerve area, the sensitivity and specificity for discrimination were somewhat lower than for cervical nerve discrimination alone (see Tables 1, 2). Apparently, the variation in nerve tissue composition observed in the histology shown in Fig. 2 requires fine tuning of the classification algorithm. To what extent this variation in composition affects the nerve discrimination for nerves at other locations in the body has to be further investigated.

To ensure that the correct tissue type was measured, two experienced anesthesiologists with the assistance of an anatomist positioned the optical needle on the exposed tissues. The exposed setting with visual confirmation was considered to be the best gold standard, as opposed to ultrasound where the needle tip/nerve visualization is prone to errors. Special attention was given to measure pure tissue types which was sometimes challenging for thin structures such as sliding fat and when placing the needle on a nerve that could slide away. Despite these difficulties, the errors that might be introduced by these measurement inaccuracies will have limited influence on the final classification accuracy due to the large dataset we acquired.

Comparing STS (sensitivity around 90 %) with other needle guidance techniques shows a significant improvement in nerve detection compared to paresthesia (sensitivity of 40 %) [6] and nerve stimulation (sensitivity up to 75 %) [7]. Another advantage of the STS method is that the detection distance (determined by the distance between fiber ends) is on the order of a millimeter, allowing more accurate placement near the nerve compared to the other methods where, even when the needle detects nerve, the exact distance is not well known. Both improvements compared to current practice may contribute to safer loco-regional blocks in regional anesthesia.

From a clinical point of view, the most vulnerable and important structure of the nerve is the nerve fascicle itself. During the administration of loco-regional anesthesia, nerve-fascicles can be damaged directly or indirectly. For instance, direct needle trauma by cutting the fascicle, pressure of injected fluid in the fascicle, overstretching of the nerve due to fluid volume or manipulation of surrounding tissue during the procedure, or indirect trauma due to ischemia by damaging the supplying blood vessel, can occur. The cause of damage, but also the site of damage, impacts the consequences. Proximal damage causes more neurological deficit than distal damage. Also, the proximal part of the nerve is more prone to damage than the distal area. In an ideal situation, the nerves should be detected right on time, the injection of fluids should not cause damage, the spread of the anesthetic fluid is perfect and the onset time of the block is fast. Several ideal needle placements are theoretically possible. One could be just outside the nerve- structure, e.g. outer epineurium. In case of proximal nerve roots or cases of nearly mono-fascicular nerves this would be achievable. Another one could be inside the epineurium, e.g. intra-epineurally or parafascicular, but not intrafascicular. This needle position may be an option in more peripheral nerves with a lot of connective tissue and fat inside the epineurium, where the risk of damaging a fascicle is probably lower.

This study shows that employing STS at the tip of the needle is a promising technique that may improve accurate needle placement near nerve structures in regional anesthesia. In particular penetration of the needle into the nerve-fascicle could be avoided. However, before this can be implemented in real clinical practice, further validation is required where the technique is investigated in an in vivo setting where the different tissue structures are approached percutaneously. This will be part of future investigations.

## Abbreviations

CART: classification and regression tree
C-roots: cervical nerve roots
Fmie: mie-to-total reduce scattering coefficient
FN: false negative
FP: false positive
F/(W + F): fat to water and fat ratio
Hb: deoxygenated-hemoglobin
HbO2: oxygenated-hemoglobin
StO2: blood oxygen saturation
MCC: Matthews correlation coefficient
PC: principal components
PCA: principal component analysis
PLS-DA: partial least-squares discriminant analysis
STS: spectral tissue sensing
TN: true negative
TP: true positive
SENS: sensitivity
SPEC: specificity
SVM: support vector machine
US: ultrasound
